# miR-9a-5p Protects Ischemic Stroke by Regulating Oxidative Stress and Mitochondrial Autophagy

**DOI:** 10.1155/2023/5146305

**Published:** 2023-02-17

**Authors:** Chunli Ma, Qing Gao, Li Zhang, Geng Wu, Chao Li, Jun Chen, Yuxuan Fu, Lei Yang

**Affiliations:** ^1^Department of Neurology, The Second Affiliated Hospital of Mudanjiang Medical University, Mudanjiang, 157010 Heilongjiang, China; ^2^Clinical Skills Center, The First Clinical Medical College of Mudanjiang Medical University, Mudanjiang, 157011 Heilongjiang, China; ^3^Department of Scientific Research, Hongqi Hospital Affiliated to Mudanjiang Medical University, Mudanjiang, 157011 Heilongjiang, China; ^4^Department of Neurology, Hongqi Hospital Affiliated to Mudanjiang Medical University, Mudanjiang, 157011 Heilongjiang, China; ^5^Department of Histology and Embryology, Mudanjiang Medical University, Mudanjiang, 157011 Heilongjiang, China; ^6^Department of Orthopaedics, Hongqi Hospital Affiliated to Mudanjiang Medical University, Mudanjiang, 157011 Heilongjiang, China

## Abstract

**Purpose:**

Present research is aimed at exploring the effect of miR-9a-5p on mitochondrial autophagy and alleviating cellular oxidative stress injury in ischemic stroke.

**Methods:**

SH-SY5Y cells were cultured with oxygen-glucose deprivation/reoxygenation (OGD/R) to simulate ischemia/reperfusion. The cells were treated in an anaerobic incubator (95% N_2_, 5% CO_2_) for 2 h and then reoxygenated in the normoxic condition for 24 h with 2 ml of normal medium. Cells were transfected with miR-9a-5p mimic/inhibitor or negative control. The RT-qPCR assay was utilized to measure the mRNA expression. Western blot was utilized to evaluate the protein expression. The CCK-8 assay was conducted to detect cell viability. Flow cytometry was applied to examine apoptosis and the cell cycle. The ELISA assay was applied to measure the contents of SOD and MDA in mitochondria. Autophagosomes were observed via electron microscopy.

**Results:**

By comparison with the control group, the miR-9a-5p expression in the OGD/R group obviously declined. Mitochondrial crista breaks, vacuole-like changes, and increased autophagosome formation were observed in the OGD/R group. OGD/R injury enhanced oxidative stress damage and mitophagy. When transfected with the miR-9a-5p mimic, mitophagosome production of SH-SY5Y cells decreased and oxidative stress injury was inhibited. However, the miR-9a-5p inhibitor obviously increased mitophagosome production and enhanced oxidative stress injury.

**Conclusion:**

miR-9a-5p protects against ischemic stroke by inhibiting OGD/R-induced mitochondrial autophagy and alleviating cellular oxidative stress injury.

## 1. Introduction

The main causes of ischemic stroke are cerebral ischemic injury caused by stenosis or occlusion of carotid arteries that provide blood, and insufficient blood supply to the brain [1–3]. It was found that autophagy is activated during cerebral ischemia and acts on other relevant pathological processes by the clearance of damaged mitochondria [4]. Mitophagy refers to the process in which cells selectively remove damaged or dysfunctional mitochondria by autophagy regulation mechanism. Under the regulation of autophagy-related proteins, the exfoliated ER membrane or Golgi body membrane is combined with damaged mitochondria to form autophagosomes with a bilayer membrane structure, gradually fuses with the lysosome to form autophagyosome, and then degrades the wrapped mitochondrial enzymes, which is important for maintaining cell survival [5, 6].

Oxidative stress is a state in which the physiological balance between oxidants and antioxidants is disrupted, biasing the balance towards the oxidant, thus causing potential damage to the body. Oxidative stress has been recognized as an important mechanism of injury in multiple diseases [7]. The pathophysiology of ischemic stroke is a complex process, with increasing free radicals and reactive oxygen species, and decreased inactivation of antioxidant enzymes and protective antioxidants, leading to the failure of natural defense mechanisms to protect neurons. Oxidative stress and its associated inflammatory response, autophagy, and apoptosis are the key to participate in nerve damage [8]. In cerebral ischemia, the balance between the oxidative system and the antioxidant system is destroyed, and the brain and immune cells produce reactive oxygen species and stimulate endothelial cells to cause oxidative stress. Meanwhile, within minutes of ischemic hypoxia, a complex molecular cascade of events follows, such as neuronal depolarization, increased Ca^2+^ influx, adenosine triphosphate depletion, and the excitatory neurotransmitter glutamate release, leading to nicotinamide adenine dinucleotide phosphate oxidase signaling activation and mitochondrial dysfunction, further aggravating oxidative stress.

The miRNAs participate in various pathophysiological processes that are closely associated with apoptosis, proliferation, and ontogeny [9]. MicroRNAs (miRNAs) are noncoding single-stranded RNA with a length of about 19-25 bases. By binding to the recognition region in the 3′UTR of the targeted miRNA, miRNAs can directly inhibit the expression of the target gene, thereby promoting mRNA degradation. Numerous studies have confirmed that miRNAs can play a role after ischemic stroke, such as promoting nerve repair, angiogenesis, and inhibiting neuronal apoptosis [10–12]. In the past few years, many studies have emphasized the interaction between miRNAs and autophagy in tumors, cerebral ischemia, neurodegenerative diseases, and other diseases [13–15]. miR-9a-5p is a rich noncoding RNA expressed in the central nervous system. Under physiological conditions, it participates in axonal extension, synaptic development, neurogenesis, and angiogenesis [16]. Furthermore, miR-9a-5p participate in the process of neurodegenerative diseases under pathological conditions. Other studies have shown that miR-9a-5p enhanced neuronal death during ischemia and demonstrated that miR-9a-5p could downregulate the expression of autophagy-related protein ATG5 [17]. However, the effect and mechanism of miR-9a-5p on oxidative stress and mitochondrial autophagy in ischemic stroke are still unclear.

This paper was aimed at investigating the role and potential mechanism of miR-9a-5p in oxidative stress and mitochondrial autophagy of ischemic stroke.

## 2. Methods

### 2.1. OGD/R Model

Human SH-SY5Y cells were maintained at 37°C and 5% CO_2_ in DMEM with penicillin, streptomycin, glutamine, and 10% FBS. After PBS washing of the cells, 2 ml of sugar-free DMEM medium was added. The cells were treated in an anaerobic incubator (95% N_2_, 5% CO_2_) for 2 h and then reoxygenated in the normoxic condition for 24 h with 2 ml of normal medium. Cells were transfected at 100 pmol/l with miR-9a-5p mimic/inhibitor or negative control via Lipofectamine 2000 (Invitrogen, USA).

### 2.2. RT-qPCR Assay

Total RNA was extracted from each group using the TRIzol reagent, and RNA was reverse transcribed into cDNA via a reverse transcription kit. qPCR was performed via the SYBR Green I dye method according to the instructions.

### 2.3. Western Blot

Lysates were added to cells to extract proteins. The supernatant was taken to detect the protein concentration via BCA. SDS-PAGE electrophoresis was performed to isolate proteins, which was transferred to a nitrocellulose membrane later. The rapid blocking liquid was closed at room temperature for 15 min. Proteins were incubated overnight at 4°C with a primary antibody. After washing, proteins were incubated with the secondary antibody for 1 h at room temperature. Then, membranes were color rendered via an ECL reagent and imaged in a gel-imaging system.

### 2.4. CCK-8 Assay

Cells were seeded in 96-well plates at 2 × 10^3^/well. According to the instructions of the CCK-8 cell viability detection kit, 100 *μ*l of CCK-8 of working solution prepared with DMEM-F12 was added. The 96-well plates were incubated in the incubator for 4 h, and the absorbance was measured at 450 nm.

### 2.5. Flow Cytometry

Cell apoptosis was evaluated using an Annexin V-FITC/PI apoptosis kit (Multisciences, China). 500 *μ*l of 1x binding buffer was used to overhang 2 × 10^5^ cells. 5 *μ*l of annexin V-FITC and 10 *μ*l of PI were added into each tube and incubated at 25°C without light for 5 min. 1 × 10^6^ cells were fixed overnight in 70% ethanol at -20°C and then incubated with a DNA-holding solution in the dark for 30 minutes. The cell cycle was detected via flow cytometry.

### 2.6. Measurement of ROS

DCFH-DA was added to the cells and incubated at 37°C for 20 min. The cells were washed three times with a serum-free cell culture medium. The cells were collected and detected via flow cytometry.

### 2.7. Mitochondrial Membrane Potential (MMP)

Cells were resuspended in a DMEM medium. 0.5 ml of JC-1 staining working solution was added to the cell suspension and mixed well. The cells were incubated in the incubator at 37°C for 20 min. After centrifugation at 4°C and 600 g for 3 min, the cells were washed thoroughly using 1 ml of JC-1 staining buffer. The cells were resuspended with the JC-1 staining buffer and analyzed using flow cytometry.

### 2.8. ELISA Assay

The contents of SOD and MDA in mitochondria of each group were measured according to the instructions of the biochemical kit.

### 2.9. Statistical Analysis

SPSS 22.0 software was utilized to analyze data. Comparison between groups was made using one-way ANOVA. *P* < 0.05 was set as the statistical threshold.

## 3. Results

### 3.1. miR-9a-5p Expression Was Downregulated in the OGD/R Model

**Figure 1:**
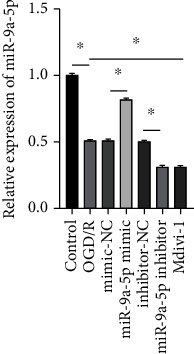
miR-9a-5p expression was downregulated in the OGD/R model. miR-9a-5p expression was measured via RT-qPCR. ^∗^*P* < 0.05.

As shown in Figure 1, the expression of miR-9a-5p in the OGD/R group decreased markedly in contrast to that in the control group. miR-9a-5p mimic improved the expression of miR-9a-5p while the expression of miR-9a-5p in the miR-9a-5p inhibitor group was observably lower. In addition, the expression of miR-9a-5p in the Mdivi-1 group was significantly reduced in contrast to that in the OGD/R group.

### 3.2. miR-9a-5p Promotes the Cell Proliferation and Inhibits Apoptosis after OGD/R Injury

**Figure 2:**
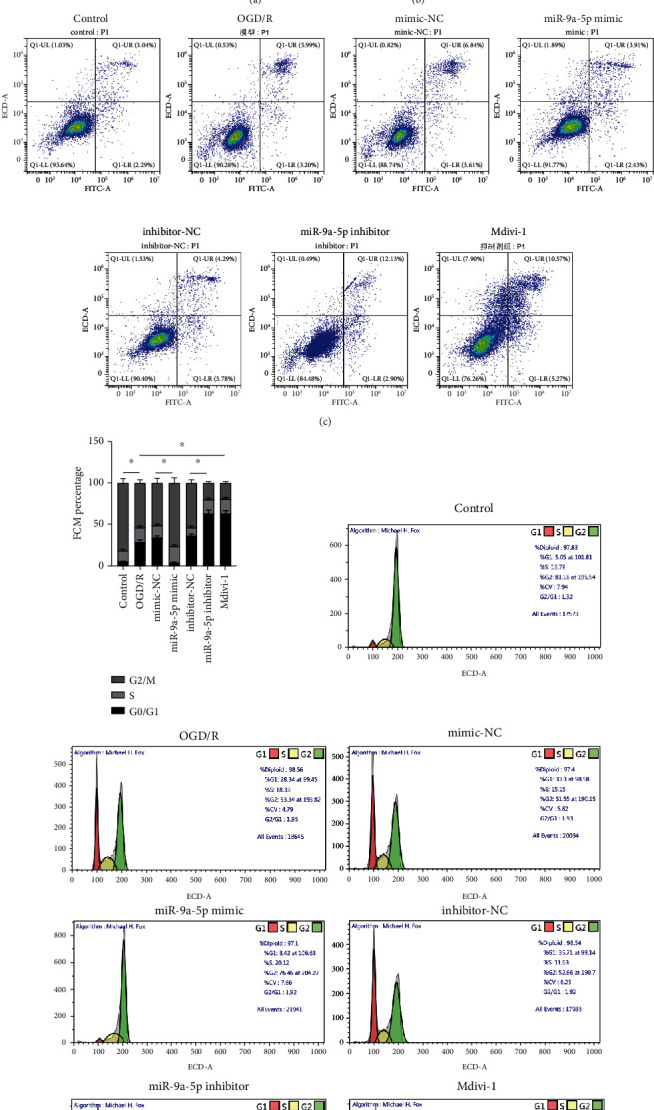
miR-367-3p promotes the cell proliferation and inhibits apoptosis after OGD/R injury. (a) Cell proliferation was detected via CCK-8 assay. (b, c) Cell apoptosis was evaluated via flow cytometry. (d) Cell cycle was detected by flow cytometry. ^∗^*P* < 0.05.

In contrast to the control group, cell proliferation was obviously inhibited in the OGD/R group (Figure 2(a)). The miR-9a-5p mimic obviously raised cell proliferation while the miR-9a-5p inhibitor markedly inhibited cell proliferation. In contrast to the OGD/R group, cell proliferation was obviously inhibited in the Mdivi-1 group. In contrast to the control group, cell apoptosis was obviously raised in the OGD/R group (Figures 2(b) and 2(c)). The miR-9a-5p mimic observably decreased cell apoptosis, while the miR-9a-5p inhibitor markedly increased cell apoptosis. In contrast to the OGD/R group, cell apoptosis was obviously raised in the Mdivi-1 group. In contrast to the control group, the proportion of the G0/G1 phase of the cell cycle increased while the proportion of the G2/M phase decreased in the OGD/R group (Figure 2(d)).

### 3.3. miR-9a-5p Alleviates Oxidative Stress after OGD/R Injury

**Figure 3:**
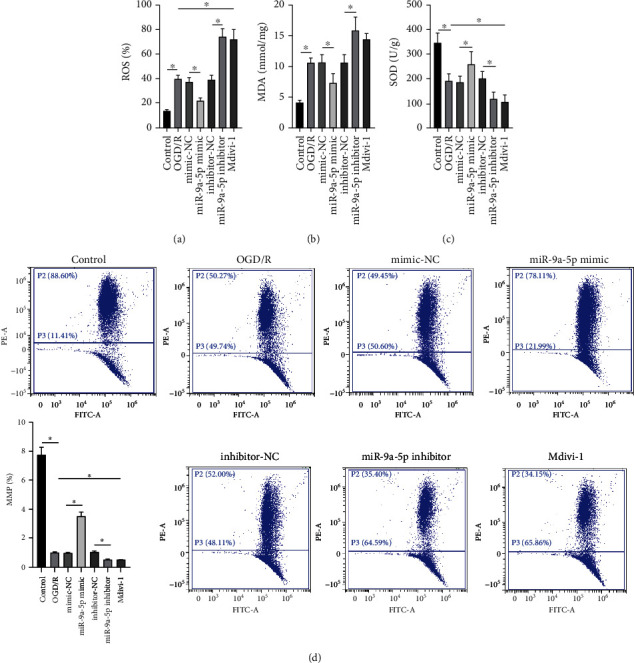
miR-9a-5p alleviates oxidative stress after OGD/R injury. (a) ROS content was detected via flow cytometry. (b) The MDA level was measured via ELISA assay. (c) The SOD activity was evaluated via ELISA assay. (d) MMP was detected via JC-1. ^∗^*P* < 0.05.

In contrast to the control group, ROS was markedly raised in the OGD/R group (Figure 3(a)). In contrast to the negative control group, ROS in the miR-9a-5p mimic group was obviously inhibited but was markedly increased in the miR-9a-5p inhibitor group. In contrast to the OGD/R group, ROS was obviously raised in the Mdivi-1 group. In contrast to the control group, MDA was observably raised in the OGD/R group (Figure 3(b)). In contrast to the negative control group, MDA in the miR-9a-5p mimic group was obviously decreased but was markedly increased in the miR-9a-5p inhibitor group. In contrast to the OGD/R group, MDA was obviously raised in the Mdivi-1 group. In contrast to the control group, SOD was observably inhibited in the OGD/R group (Figure 3(c)). In contrast to the negative control group, SOD in the miR-9a-5p mimic group was observably raised but was markedly inhibited in the miR-9a-5p inhibitor group. In contrast to the OGD/R group, SOD was obviously inhibited in the Mdivi-1 group. In contrast to the control group, MMP in the OGD/R group was observably decreased (Figure 3(d)). The miR-9a-5p mimic significantly increased MMP, while the inhibitor significantly decreased MMP. In addition, in contrast to the OGD/R group, MMP was obviously inhibited in the Mdivi-1 group.

### 3.4. miR-9a-5p Regulates Mitochondrial Autophagy after OGD/R Injury

**Figure 4:**
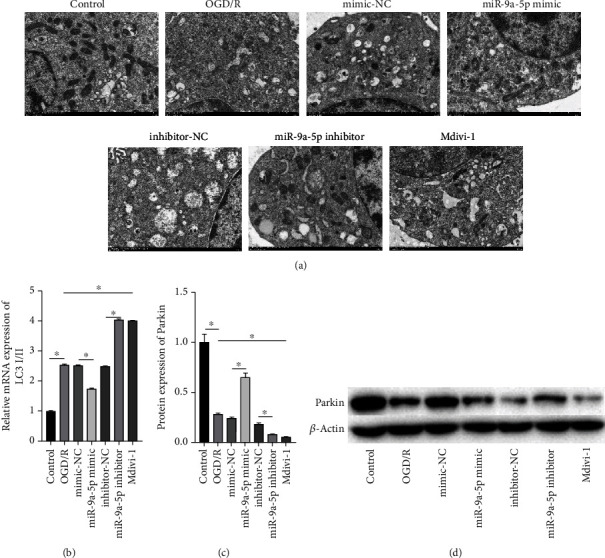
miR-9a-5p regulates mitochondrial autophagy after OGD/R injury. (a) Autophagosomes were observed via electron microscopy. (b) The mRNA expression of IC3 I/II was examined via RT-qPCR. (c, d) Protein expression of Parkin was detected via western blot. ^∗^*P* < 0.05.

As shown in Figure 4(a), transmission electron microscopy showed that the mitochondria of control groups were clear and complete, and only a small number of autophagosomes were formed. Mitochondrial crista breaks, vacuole-like changes, and increased autophagosome formation were observed in the OGD/R group. In contrast to the OGD/R group, mitochondria swelling, crista rupture, and vacuole-like changes were alleviated in the miR-9a-5p mimic group, and autophagosome formation was reduced. In the miR-9a-5p inhibitor group, mitochondria swelling, crista rupture, and vacuole-like changes were aggravated, and autophagosome formation was increased. As shown in Figure 4(b), in contrast to the control group, the mRNA expression of IC3 I/II in the OGD/R group was increased obviously. In contrast to the negative control group, the mRNA expression of IC3 I/II in the miR-9a-5p mimic group was markedly lower but was obviously higher in the miR-9a-5p inhibitor group. In addition, in contrast to the OGD/R group, the mRNA expression of IC3 I/II in the Mdivi-1 group was significantly raised. In contrast to the control group, the protein expression of Parkin in the OGD/R group was decreased (Figures 4(c) and 4(d)). In contrast to the negative control group, the miR-9a-5p mimic markedly raised the protein expression of Parkin while the miR-9a-5p inhibitor decreased it. In addition, in contrast to the OGD/R group, the protein expression of Parkin in the Mdivi-1 group was significantly decreased.

## 4. Discussion

Stroke is a common multiple acute cerebrovascular disease, in which ischemic stroke is the most common stroke subtype, accounting for 80%-85% of all stroke cases. Stroke ranks second among all major diseases, and stroke is characterized by acute episodes, high mortality, and many complications [18]. China has gradually entered an aging population stage, and stroke has become the leading cause of death. Stroke has a high rate of mortality and disability, and the rapid oedema occurs with space occupation after cerebral infarction, leading to intracranial hypertension and brain hernia, leading to death [19].

Mitochondria are the energy center, and numerous redox reactions occur in the inner mitochondrial membrane, so the mitochondria are the main source of ROS production. Poststroke reperfusion supplementation of nutrients and oxygen reactivates mitochondrial aerobic respiration and leads to ROS [20], a major source of intracellular ROS. This study found that the level of ROS increased and oxidative stress enhanced in SH-SY5Y cells after OGD/R injury (including MDA increase and SOD decrease). In addition, miR-9a-5p could alleviate oxidative stress in SH-SY5Y cells after OGD/R injury. ROS is the main substance-mediating oxidative stress response, and protein molecules are the main target molecules of ROS. ROS modifies the protein side chain by splitting or crosslinking the peptide chain, interacts with the active center, or changes the protein 1-4 structure, resulting in protein denaturation or protease inactivation. These changes in turn lead to a wide range of functional changes, such as abnormal downstream enzymatic reaction, decreased proteolytic capacity, and accumulation of damaged proteins, and ultimately lead to apoptosis and necrosis [21]. ROS is a trigger for PINK1/Parkin-mediated mitophagy and enables mitochondrial renewal through this process. The present study demonstrated that OGD/R injury obviously decreased Parkin expression while miR-9a-5p enhanced Parkin expression. The study by Wang et al. also found that electroacupuncture treatment significantly increased the expression levels of Drp1 and Parkin, promoted the translocation of Parkin and LC3 to the mitochondria, and effectively reduced the levels of ROS and MDA, while Mdivi-1 could reverse these effects [22]. The PINK1/Parkin pathway is the main pathway mediating mitophagy, and PINK1 is a cell mitochondrial outer membrane protein; under normal circumstances, its outer membrane maintains low levels, when mitochondrial damage results in depolarization. PINK1 is heavily concentrated in the mitochondrial outer membrane and recruits cytoplasmic Parkin protein, promotes Parkin protein transfer to mitochondria which is activated by phosphorylation, and promotes mitophagy [23].

Excessive ROS leads to mitochondrial depolarization, inducing mitophagy [24, 25], and ROS-mediated mitophagy is a negative feedback mechanism that reduces ROS production by removing impaired mitochondria. Mitochondrial autophagy is an adaptive regulatory mechanism for cells to clear damaged mitochondria, which is closely related to the occurrence of a variety of diseases and the development of pathological processes. Research has confirmed that there is a decrease in mitochondrial autophagy in Alzheimer's disease, Huntington's disease, Parkinson's disease, and so on [26, 27]. Studies have confirmed that autophagy activation protects against cerebral ischemic nerve cells caused by hypoxia and that autophagy inhibition by oxygen-glucose deprivation for 2 h can aggravate brain injury. This study found that OGD/R injury induced mitochondrial autophagy while miR-9a-5p inhibited mitochondrial autophagy in SH-SY5Y cells after OGD/R injury. The characteristic that nerve cells depend on mitochondria for energy supply leads to its abnormal sensitivity to the selective clearance of mitochondria, which also makes the dysfunction of mitophagy play an important role in cerebral ischemia-reperfusion injury [28]. Excessive ROS produced after cerebral ischemia-reperfusion can increase LC3-II expression, thereby activating mitochondrial autophagy, clearing damaged mitochondria and excessive ROS.

In this study, SH-SY5Y cells were treated with OGD/R injury, and the cell viability in the OGD/R group was lower than that in the control group, and the residual mitochondrial melanin and membrane autophagosomes were increased after autophagy. It has been shown that ROS, as cell signaling molecules, could activate autophagosome formation and autophagic degradation, and autophagy can also regulate the levels of ROS through the chaperone-mediated autophagy (CMA) pathway, P62 transmission pathway, and mitotic pathway [29]. Therefore, activating autophagy to clear the mitochondria and proteins of reactive oxygen species damage may be an important means to protect the cells from oxidative stress injury during cerebral ischemia and reperfusion injury.

It is concluded that miR-9a-5p protected ischemic stroke by inhibiting OGD/R-induced mitochondrial autophagy and alleviating cellular oxidative stress injury. Due to the limitations of cell experiments, further animal experiments will be carried out later to verify the effect of miR-9a-5p on oxidative stress and mitophagy in ischemic stroke and to provide a more reliable basis for clinical treatment.

## Data Availability

Data to support the findings of this study is available on reasonable request from the corresponding author.
